# The *in vivo* function of TIGAR, a p53 target gene that regulates glucose metabolism

**DOI:** 10.1186/2049-3002-2-S1-P15

**Published:** 2014-05-28

**Authors:** Eric Cheung, Pearl Lee, Celia Berkers, Karen Blyth, Owen Sansom, Karen Vousden

**Affiliations:** 1 The Beatson Institute for Cancer Research, Glasgow, UK

## 

The p53 tumour suppressor inhibits tumour development via various mechanisms such as apoptosis, inhibition of proliferation or the activation of senescence. Recently, several studies have indicated a novel role of p53 in the regulation of energy metabolism. Previously we have discovered TIGAR, a p53 target gene that acts as a fructose-2,6-bisphosphatase. TIGAR would therefore be predicted to redirect glucose from the glycolytic pathway to secondary pathways such as the pentose phosphate pathway (PPP). Indeed, TIGAR can promote NADPH production to generate reduced glutathione for protection against ROS. In order to understand the function of TIGAR *in vivo*, we generated TIGAR deficient mice. We have determined a critical role of TIGAR in rapidly proliferating tissue, either for repair after damage or during tumor development. These studies support a role for TIGAR in maintaining both antioxidant activity and nucleotide synthesis, both generated through the PPP. We are now also investigating the role of TIGAR in other metabolic pathways such as the hexosamine biosynthesis pathway, and in other animal models of cancer.

